# Supplementary material for: Teratogenic, Oxidative Stress and Behavioural Outcomes of Three Fungicides of Natural Origin (Equisetum arvense, Mimosa tenuiflora, Thymol) on Zebrafish (Danio rerio)
Source: Toxics. 2021 Jan 9;9(1):8. doi: 10.3390/toxics9010008 (PMC7827758; doi:10.3390/toxics9010008)
Supplement: Supplementary file 1 [file toxics-09-00008-s001.pdf]

Supplementary Materials: Teratogenic, Oxidative Stress and Behavioural Outcomes of Three Fungicides of Natural Origin (*Equisetum arvense, Mimosa tenuiflora,* Thymol) on Zebrafish (*Danio rerio*)

Raquel Vieira [1], Carlos Venâncio [1,2] and Luís Félix [1,3,4,*]

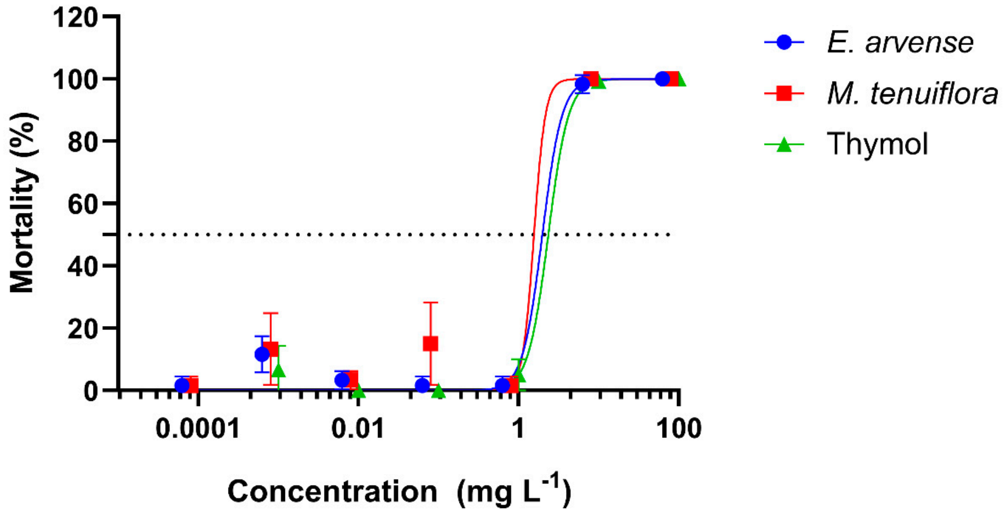

Figure S1.

Table S1. Sublethal effects of the different exposures on the development of zebrafish embryos.

Phyto-fungicide	Group	24 hpf			48 hpf			72 hpf
		Tail Not Detached	Head Not Detached	Somite Not Formed	Eyes Not Developed	Otoliths Not Developed	Blood Circulation Not Visible	Oedema Presence (Yolk Sac and Cardiac)
Equisetum arvense extract	Ctrl	nd	nd	nd	nd	nd	nd	nd
	E1	nd	nd	nd	nd	nd	nd	nd
	E2	nd	nd	nd	nd	nd	nd	nd
	E3	nd	nd	nd	nd	nd	nd	nd
Mimosa tenuiflora extract	Ctrl	nd	nd	nd	nd	nd	nd	nd
	M1	nd	nd	nd	nd	nd	nd	nd
	M2	nd	nd	nd	nd	nd	nd	nd
	M3	nd	nd	nd	nd	nd	nd	nd
Thymol	Ctrl	nd	nd	nd	nd	nd	nd	0.00 (0.00–5.00)
	T1	nd	nd	nd	nd	nd	nd	0.00 (0.00–10.0)
	T2	nd	nd	nd	nd	nd	nd	0.00 (0.00–10.0)
	T3	nd	nd	nd	nd	nd	nd	0.00 (0.00–0.00)

Non-parametric data presented as median and interquartile range of five independent replicates. Statistical analysis was performed using the Kruskal–Wallis test followed by Dunn's test ($p < 0.05$). nd—not detected

Table S2. Biochemical parameters evaluated at 98 hpf in zebrafish embryos exposed to *Equisetum arvense* extract.

Test Parameter	Exposure Group				Statistical Test	*p*-Value
	Ctrl	E1	E2	E3		
ROS (µmol DCF mg protein^{-1})	64.5 ± 16.1	83.7 ± 20.8	66.1 ± 15.8	70.9 ± 20.8	F(3,16) = 1.106	0.376
SOD (U mg protein^{-1})	105.6 (103.0–143.4)	142.0 (126.8–153.8)	122.9 (106.6–139.3)	119.8 (109.1–147.6)	X^2(3) = 3.600	0.308
CAT (U mg protein^{-1})	0.5 (0.4–0.6)	0.3 (0.1–0.4)	0.2 (0.2–0.4)	0.3 (0.2–0.5)	X^2(3) = 5.094	0.165
GPx (nmol NADPH min^{-1} mg protein^{-1})	**3.2 (2.0–9.3)** ^{a,b}	**4.5 (2.6–6.3)** ^{a,b}	**9.4 (7.5–11.3)** ^{a}	**1.6 (1.1–5.4)** ^{b}	**X^2(3) = 8.623**	**0.035**
GR (nmol NADPH min^{-1} mg protein^{-1})	**9.1 (5.5–12.7)** ^{a,b}	**21.0 (17.7–26.0)** ^{b}	**17.4 (12.5–23.5)** ^{a,b}	**6.1 (2.3–11.9)** ^{a}	**X^2(3) = 12.27**	**0.007**
GSH (µmol GSH mg protein^{-1})	28.1 ± 11.7	14.9 ± 4.0	19.3 ± 9.0	23.5 ± 2.1	F(3,15) = 2.701	0.083
GSSG (µmol GSSG mg protein^{-1})	28.2 (17.1–33.6)	12.8 (9.45–17.8)	13.9 (8.65–21.0)	20.2 (15.9–23.7)	X^2(3) = 7.000	0.072
GSH:GSSG	1.3 (1.0–1.4)	1.1 (1.0–1.3)	1.4 (1.1–1.5)	1.3 (1.1–1.4)	X^2(3) = 3.076	0.380
GST (µmol CDNB min^{-1} mg protein^{-1})	3.4 ± 1.0	2.2 ± 0.5	2.5 ± 0.8	2.9 ± 0.5	F(3,16) = 2.414	0.105
CarE (nmol 4-nitrophenol min^{-1} mg protein^{-1})	14.1 ± 5.5	18.4 ± 3.7	17.0 ± 3.63	14.2 ± 3.3	F(3,16) = 2.000	0.155
LPO (µmol MDA mg protein^{-1})	0.6 (0.4–1.1)	0.8 (0.4–1.1)	1.2 (0.7–1.3)	1.0 (0.8–1.1)	X^2(3) = 2.657	0.448
AChE (nmol TNB min^{-1} mg protein^{-1})	**75.0 ± 16.3** ^{a}	**167.8 ± 21.7** ^{b}	**146.6 ± 80.6** ^{a,b}	**157.9 ± 049.4** ^{a,b}	**F(3,16) = 3.526**	**0.039**
LDH (nmol NADH min^{-1} mg protein^{-1})	133.3 (105.4–145.1)	133.6 (109.1–150.6)	117.8 (110.6–149.0)	85.5 (78.1–119.5)	X^2(3) = 4.687	0.196

Data from five independent replicates expressed as mean ± SD for parametric data distribution or median and interquartile range for non-parametric data. Statistical analysis was performed using one-way ANOVA followed by Tukey's multiple-comparison test or Kruskal-Wallis followed by Dunn's test. Different lowercase letters indicate significant differences between groups ($p < 0.05$).

Table S3. Biochemical parameters evaluated at 98 hpf in zebrafish embryos exposed to *Mimosa tenuiflora* extract.

Test Parameter	Exposure Group				Statistical Test	*p*-Value
	Ctrl	M1	M2	M3		
ROS (µmol DCF mg protein^{-1})	28.6 ± 14.0	11.5 ± 3.8	22.3 ± 8.3	27.9 ± 8.4	F(3,16) = 2.101	0.140
SOD (U mg protein^{-1})	169.5 (104.3–278.3)	110.3 (78.4–222.2)	241.7 (180.5–278.4)	131.6 (126.3–204.4)	X^2(3) = 4.509	0.212
CAT (U mg protein^{-1})	0.2 ± 0.1	0.3 ± 0.1	0.3 ± 0.1	0.3 ± 0.1	F(3,16) = 0.571	0.642
GPx (nmol NADPH min^{-1} mg protein^{-1})	5.6 ± 4.3	14.2 ± 7.1	13.9 ± 5.9	7.9 ± 4.6	F(3,16) = 2.960	0.063
GR (nmol NADPH min^{-1} mg protein^{-1})	19.1 ± 9.5	16.6 ± 8.1	20.7 ± 15.0	14.6 ± 6.0	F(3,16) = 0.344	0.794
GSH (µmol GSH mg protein^{-1})	5.5 (4.3–8.7)	7.1 (6.6–21.2)	4.5 (4.2–8.8)	6.9 (5.0–13.8)	X^2(3) = 5.583	0.134
GSSG (µmol GSSG mg protein^{-1})	6.1 (3.4–7.5)	4.2 (2.4–10.3)	2.0 (1.9–6.8)	5.9 (2.9–9.4)	X^2(3) = 2.154	0.541
GSH:GSSG	1.2 (0.8–1.6)	2.4 (1.5–3.1)	2.1 (1.5–2.3)	1.8 (1.0–1.9)	X^2(3) = 7.834	0.050
GST (nmol CDNB min^{-1} mg protein^{-1})	31.7 (22.9–54.5)	26.6 (10.1–28.4)	32.2 (11.5–34.6)	24.5 (10.9–36.2)	X^2(3) = 3.537	0.316
CarE (nmol 4-nitrophenol min^{-1} mg protein^{-1})	7.6 (6.8–8.9)	7.2 (4.5–8.5)	7.6 (4.1–7.9)	7.6 (5.9–10.0)	X^2(3) = 1.196	0.754
LPO (µmol MDA mg protein^{-1})	0.4 (0.3–0.4)	0.4 (0.3–1.1)	0.2 (0.2–0.4)	0.4 (0.3–0.6)	X^2(3) = 3.754	0.289
AChE (nmol TNB min^{-1} mg protein^{-1})	32.7 ± 7.5	42.8 ± 3.1	44.4 ± 14.2	53.3 ± 22.6	F(3,16) = 1.829	0.183
LDH (nmol NADH min^{-1} mg protein^{-1})	109.2 ± 22.3	83.9 ± 24.1	101.6 ± 21.8	92.0 ± 42.5	F(3,16) = 0.730	0.549

Data from five independent replicates expressed as mean ± SD for parametric data distribution or median and interquartile range for non-parametric data. Statistical analysis was performed using one-way ANOVA followed by Tukey's multiple-comparison test or Kruskal-Wallis followed by Dunn's test.

Table S4. Biochemical parameters evaluated at 98 hpf in zebrafish embryos exposed to Thymol.

Test Parameter	Exposure Group				Statistical Test	*p*-Value
	Ctrl	M1	M2	M3		
ROS (μmol DCF mg protein^{-1})	23.8 ± 4.0	23.6 ± 5.0	18.5 ± 5.6	29.0 ± 7.1	F(3,16) = 3.051	0.059
SOD (U mg protein^{-1})	138.1 ± 76.0	179.8 ± 70.1	70.9 ± 18.0	110.9 ± 72.3	F(3,16) = 2.589	0.089
CAT (U mg protein^{-1})	0.5 ± 0.2	0.3 ± 0.2	0.4 ± 0.2	0.2 ± 0.0	F(3,16) = 2.133	0.136
GPx (nmol NADPH min^{-1} mg protein^{-1})	8.3 (3.2–13.5)	18.9 (11.7–26.2)	10.8 (7.5–13.8)	12.3 (9.8–14.7)	$X^2(3) = 7.286$	0.063
GR (nmol NADPH min^{-1} mg protein^{-1})	8.9 (6.9–12.8)	18.5 (11.9–25.0)	16.0 (9.8–16.3)	16.2 (15.4–19.9)	$X^2(3) = 7.844$	0.066
GSH (μmol GSH mg protein^{-1})	10.6 (5.5–17.4)	11.7 (7.6–18.1)	24.9 (16.9–38.6)	14.8 (8.8–22.1)	$X^2(3) = 6.337$	0.096
GSSG (μmol GSSG mg protein^{-1})	10.7 ± 2.9	5.9 ± 3.7	11.7 ± 3.7	8.1 ± 3.2	F(3,16) = 3.048	0.059
GSH:GSSG	**1.0 ± 0.3** [a]	**2.3 ± 0.4** [b]	**2.3 ± 0.5** [b]	**1.8 ± 0.4** [b]	**F(3,16) = 11.21**	**<0.001**
GST (nmol CDNB min^{-1} mg protein^{-1})	**17.7 (12.2–21.9)** [a]	**23.4 (19.4–28.2)** [a,b]	**23.8 (21.6–25.1)** [a,b]	**32.6 (26.3–37.3)** [b]	**$X^2(3) = 12.60$**	**0.006**
CarE (nmol 4-nitrophenol min^{-1} mg protein^{-1})	6.7 ± 2.7	7.4 ± 4.8	5.0 ± 1.5	7.4 ± 2.7	F(3,16) = 0.654	0.592
LPO (μmol MDA mg protein^{-1})	0.6 (0.2–1.1)	0.4 (0.3–0.8)	0.8 (0.5–1.5)	0.5 (0.3–1.0)	$X^2(3) = 2.726$	0.436
AChE (nmol TNB min^{-1} mg protein^{-1})	32.5 (22.8–33.5)	30.5 (28.4–44.4)	27.8 (25.7–37.2)	33.9 (28.1–39.1)	$X^2(3) = 2.360$	0.501
LDH (nmol NADH min^{-1} mg protein^{-1})	93.4 ± 27.0	94.7 ± 12.9	88.6 ± 15.2	109.9 ± 49.6	F(3,16) = 0.474	0.705

Data from five independent replicates expressed as mean ± SD for parametric data distribution or median and interquartile range for non-parametric data. Statistical analysis was performed using one-way ANOVA followed by Tukey's multiple-comparison test or Kruskal-Wallis followed by Dunn's test. Different lowercase letters indicate significant differences between groups ($p < 0.05$).

Table S5. Exploratory behaviour of 120 hpf eleutheroembryo exposed to *Equisetum arvense* extract.

Test Parameter	Exposure Group				Statistical Test	*p*-Value
	Ctrl	E1	E2	E3		
Speed (cm min^{-1})	8.1 ± 1.9	8.2 ± 0.7	8.6 ± 0.3	7.9 ± 2.3	F(3,16) = 0.222	0.879
Distance moved (cm)	234.9 ± 106.9	297.2 ± 74.38	340.8 ± 41.8	218.1 ± 100.3	F(3,16) = 2.254	0.124
Distance to centre (cm)	1.3 ± 0.2	1.2 ± 0.1	1.2 ± 0.1	1.2 ± 0.1	F(3,16) = 0.680	0.577
Active (%)	46.5 ± 15.1	32.7 ± 5.2	31.3 ± 2.2	39.9 ± 12.0	F(3,16) = 2.429	0.103
Absolute turn angle (°)	7.7 ± 2.3	8.3 ± 0.7	9.0 ± 0.6	8.8 ± 3.0	F(3,16) = 0.460	0.714

Data from five independent replicates expressed as mean ± SD. Statistical analysis was performed using one-way ANOVA followed by Tukey's multiple-comparison test ($p < 0.05$).

Table S6. Exploratory behaviour of 120 hpf eleutheroembryo exposed to *Mimosa tenuiflora* extract.

Test Parameter	Exposure Group				Statistical Test	*p*-Value
	Ctrl	M1	M2	M3		
Speed (cm min^{-1})	7.5 ± 3.0	5.6 ± 2.1	7.5 ± 3.4	7.3 ± 2.0	F(3,16) = 0.434	0.732
Distance moved (cm)	196.0 ± 126.9	83.5 ± 62.1	152.7 ± 100.2	161.4 ± 88.0	F(3,16) = 0.965	0.439
Distance to centre (cm)	1.2 ± 0.1	1.0 ± 0.2	1.1 ± 0.1	1.1 ± 0.2	F(3,16) = 0.791	0.517
Active (%)	42.9 ± 15.1	59.9 ± 14.9	41.8 ± 18.3	54.1 ± 12.6	F(3,16) = 1.629	0.222
Absolute turn angle (°)	9.9 ± 2.4	5.7 ± 1.4	9.0 ± 4.3	6.5 ± 1.9	F(3,16) = 2.631	0.091

Data from five independent replicates expressed as mean ± SD. Statistical analysis was performed using one-way ANOVA followed by Tukey’s multiple-comparison test ($p < 0.05$).

Table S7. Exploratory behaviour of 120 hpf eleutheroembryo exposed to Thymol.

Test Parameter	Exposure Group				Statistical Test	*p*-Value
	Ctrl	T1	T2	T3		
Speed (cm min^{-1})	7.4 ± 3.0	8.1 ± 2.8	8.2 ± 2.3	7.3 ± 2.1	F(3,16) = 0.158	0.923
Distance moved (cm)	138.9 ± 107.2	155.6 ± 130.9	154.4 ± 54.5	140.3 ± 108.0	F(3,16) = 0.071	0.975
Distance to centre (cm)	1.2 ± 0.1	1.2 ± 0.1	1.2 ± 0.2	1.2 ± 0.2	F(3,16) = 0.337	0.799
Active (%)	53.2 ± 26.9	66.1 ± 26.2	38.8 ± 18.7	54.7 ± 12.4	F(3,16) = 1.310	0.306
Absolute turn angle (°)	8.3 ± 4.2	8.5 ± 4.4	7.7 ± 1.7	8.0 ± 1.2	F(3,16) = 0.065	0.978

Data from five independent replicates expressed as mean ± SD. Statistical analysis was performed using one-way ANOVA followed by Tukey’s multiple-comparison test ($p < 0.05$).
